# Incarceration as a key variable in racial disparities of asthma prevalence

**DOI:** 10.1186/1471-2458-10-290

**Published:** 2010-05-28

**Authors:** Emily A Wang, Jeremy Green

**Affiliations:** 1 Section of General Medicine, Department of Medicine, Yale University School of Medicine, New Haven, CT, USA; 2 Health Policy and Administration Program, Yale University School of Public Health, New Haven, CT, USA

## Abstract

**Background:**

Despite the disproportionate incarceration of minorities in the United States, little data exist investigating how being incarcerated contributes to persistent racial/ethnic disparities in chronic conditions. We hypothesized that incarceration augments disparities in chronic disease.

**Methods:**

Using data from the New York City Health and Nutrition Examination Study, a community-based survey of 1999 adults, we first estimated the association between having a history of incarceration and the prevalence of asthma, diabetes, hypertension using propensity score matching methods. Propensity scores predictive of incarceration were generated using participant demographics, socioeconomic status, smoking, excessive alcohol and illicit drug use, and intimate partner violence. Among those conditions associated with incarceration, we then performed mediation analysis to explore whether incarceration mediates racial/ethnic disparities within the disease.

**Results:**

Individuals with a history of incarceration were more likely to have asthma compared to those without (13% vs. 6%, p < 0.05) and not more likely to have diabetes or hypertension, after matching on propensity scores. Statistical mediation analysis revealed that increased rates of incarceration among Blacks partially contribute to the racial disparity in asthma prevalence.

**Conclusion:**

Having been incarcerated may augment racial disparities in asthma among NYC residents. Eliminating health disparities should include a better understanding of the role of incarceration and criminal justice policies in contributing to these disparities.

## Background

Incarceration has become increasingly frequent in the lives of Americans in the past two decades, with more than 2.3 million individuals behind bars[1] and an additional 8 million under the supervision of parole and probation [2]. Racial and ethnic minorities are disproportionately represented among current and former inmates [3]. For instance, while African-Americans make up 13% of the general US population, they constitute 28% of all arrests and 40% of all people held in prisons and jails, whereas Whites make up 67% of the US population and 70% of all arrests, but only 40% of all people held in state prisons or local jails [3]. Additionally, Black and Latino inmates serve longer sentences for similar crimes compared to their White counterparts.

The disproportionate minority contact with the criminal justice system has implications for individual's health both in prison and after release. Recent studies show that individuals with a history of incarceration are more likely to have asthma, diabetes, and incident hypertension compared to the general population [4-7]. While the specific reasons for increased rates of disease in this population are under investigation, researchers have identified a number of different pathways for why incarceration might augment chronic conditions. To start, prisons and jails are often poorly ventilated, dilapidated, and overcrowded--sites for transmission or exacerbation of respiratory diseases [8,9]. They offer limited opportunities for cardiovascular disease prevention through healthy diets, regular exercise, or chronic disease education and self-management skills [10,11]. Moreover, individuals released from prison face legal barriers to employment, housing, public entitlements, and educational opportunities,[12-14] which may exacerbate already limited access to community health care and medical treatment.

For these reasons, we hypothesized that disproportionate rates of incarceration among racial and ethnic minorities may play a role in contributing to racial health disparities in chronic conditions [15-17]. Black and Latino subgroups face persistent health disparities in asthma, diabetes, and hypertension,[18,19] differentials that parallel those in incarceration, suggesting that perhaps direct exposure to prison, jails, or criminal justice policies may contribute to the increased rates of asthma, diabetes, and hypertension among racial and ethnic minorities. The New York City Health and Nutrition Examination Survey (NYC HANES) provides the unique opportunity to explore incarceration as an contributory factor in racial and ethnic disparities in these health conditions in a community based sample. Using data from the NYC HANES, we examined the impact of having a history of incarceration on the prevalence of asthma, diabetes, and hypertension using both standard regression and propensity score matching techniques; empirically tested whether having a history of incarceration mediates racial and ethnic disparities in these conditions using statistical mediation analysis; and examined access to health care among those with prior incarceration.

## Methods

### Sample and Setting

Modeled after the National Health and Nutrition Examination Survey, the NYC HANES is a population-based, cross-sectional survey of non-institutionalized NYC residents ≥ 20 years old. The NYC HANES was designed to provide citywide prevalence information on conditions identifiable only through a physical examination or biologic specimen testing (i.e., hypertension or diabetes) and conditions that are not easily ascertained by a telephone survey (i.e., drug use, incarceration, and domestic violence). The study design is described in detail in previous publications [20]. Briefly, NYC HANES measured key health indicators in a sample of randomly selected community residents. A 3-stage, cluster sampling plan was used to recruit participants between June and December of 2004. Sample selection included random selection of census blocks or groups of blocks, followed by households within the selected segment, and finally study participants within selected households. The survey included a face-to-face computer assisted personal interview, private audio computer assisted self-interview for questions about drug use and incarceration, and a physical examination and laboratory testing. Of the 3047 selected eligible survey participants, 1999 individuals completed the interview for an overall response rate of 55%. All participants gave informed consent, and the study received approval from the NYC Department of Health and Mental Hygiene Institutional Review Board. This study focuses on the role of incarceration in the health of this population of 1999 individuals, among whom 160 (8%) reported a history of incarceration.

### Analytic Variables

#### History of incarceration

To assess history of incarceration, participants were asked: "Have you ever spent any time in a correctional facility, jail, prison, or detention center as an adult, that is, 18 years old or older?" A positive response to this item was categorized as having been incarcerated. Individuals who did not respond to this question were excluded from the analysis (N = 9).

#### Chronic Disease Measures

To assess asthma, we used a series of questions that captured an individual's history of asthma and measures of asthma severity. Participants who responded yes to both: "Has a doctor or other health professional ever told you that you have asthma?" and do you "still have asthma?" were categorized as currently suffering from asthma. Participants who reported currently having asthma were then asked the following measures of disease severity: "During the past 12 months have you had an episode of asthma or an asthma attack?" and "During the past 12 months have you had to visit an emergency room or urgent care clinic because of your asthma?" Diabetes was defined by self-reported disease, fasting glucose ≥ 126 mg/dL, or use of anti-diabetic medication. Hypertension was defined as a systolic blood pressure ≥ 140 mmHg, diastolic blood pressure ≥ 90 mmHg, or current use of prescribed anti-hypertension medication.

#### Potential confounders

The NYC HANES contains a variety of potential confounders allowing us to better assess the independent contribution of having a history of incarceration on the prevalence of asthma, diabetes, and hypertension. Socioeconomic status of each participant was approximated using measures of self-reported educational attainment and family income. Participants were asked: "Which of these categories best describes your total combined family income for the past 12 months?" Those who responded that their family income was less than $20,000 were defined as meeting 100% of the federal poverty line and being of low socioeconomic status [21].

For cigarette smoking, participants were categorized as current smokers if they indicated that they "smoke every day or some days." Former smokers were defined as those who reported having smoked at least 100 cigarettes in their entire life, but did not currently smoke. Use of cocaine, intravenous drugs, and excessive alcohol was ascertained with private audio computer assisted self-interview. For cocaine and intravenous drug use, participants were categorized as current users if they had used in the past 12 months, former users if they had ever used but not in the past 12 month, or never users. For alcohol use, participants were categorized as excessive alcohol consumers based on the at-risk consensus thresholds of the National Institute on Alcohol Abuse and Alcoholism, which were met if a man consumed equal to or greater than 14 drinks per week or a woman more than 7 drinks per week, where a drink was counted as 12 ounces of beer, 5 ounces of wine or 1-1/2 ounces of spirits [22]. Binge drinking was categorized as drinking more than 5 drinks on one day in the past 12 months. Intimate partner violence was measured by a positive response to the following question: "In the past 12 months have you been frightened for the safety of yourself, your children, or friends because of anger or threats of an intimate partner?"

#### Health care access

To assess health care access, participants were asked if they had a primary care provider with a series of questions, beginning with: "Is there a place that you usually go when you are sick or you need advice about your health?" Respondents who answered "yes" were then asked, "What kind of place do you go to most often: is it a clinic, doctor's office, emergency room or some other place?" Those who responded "clinic or health center" or "doctor's office or HMO" were coded as having a regular primary care provider. Participants were also asked whether they had "health insurance or some other kind of health care plan" to define insurance status.

### Analytic Methods

A challenge to estimating the true impact of incarceration on health outcomes is that studies which randomly assign individuals to incarceration are unethical and unfeasible. Observational studies are complicated by the fact that individuals who have been incarcerated are inevitably different from individuals who have never been, which may bias the estimated impact of incarceration on health outcomes. As a result, researchers have devised strategies for observational studies that approximate the design features of a randomized experiment and reduce confounding. These include statistical control with multiple regression and propensity score matching. Because multiple regression may lead to overparameterization if the number of potential confounders is large relative to the number of study units, we chose to use both methods to estimate the influence of having a history of incarceration on the prevalence of chronic diseases.

#### Propensity Score Matching

Propensity score methods use measured participant demographics and clinical and behavioral characteristics to match individuals on the basis of their likelihood to experience a treatment, in this case, their likelihood of having been incarcerated [23]. By matching on background attributes of individuals that might otherwise confound the estimated impact of incarceration on asthma, diabetes, and hypertension prevalence, we can draw inference from the comparison of similar "treated" and "non-treated" individuals. The identifying assumption is that, conditional on measured characteristics, individuals who have been incarcerated would have the same health outcomes as those who have not been incarcerated, were it not for having been incarcerated:

where Y is a health outcome of interest, D is incarceration status, 1 denotes individuals who have been incarcerated and 0 denotes those individuals who have not been incarcerated, and S is a vector of observable characteristics of individuals to which the matching estimator is applied. The propensity score is an estimate of the probability that an individual has previously been incarcerated:

with D = 1 denoting having been incarcerated and the Xs are a series of observable background attributes of the individuals. Assuming incarceration is properly modeled, using the propensity score allows us to treat incarceration as random and thus helps alleviate concerns about sample heterogeneity. In contrast to traditional regression, propensity scores often produce better adjustment of baseline differences than simply including potential confounders in a regression model for two primary reasons. First, the analyst does not have to make assumptions regarding linearity and additivity in the relationship between the individual confounders and the outcomes. Second, propensity scores create a homogenous sample, given the covariates in the model, where individuals differ only in whether they have experienced incarceration [24].

We estimated average treatment on treated (ATT) effects by first calculating the difference in mean prevalence of asthma, diabetes, and hypertension between those individuals who have and have not been incarcerated in the unmatched sample. In order to further demonstrate the robustness of our results, we matched on propensity scores using four different matching procedures (with and without common support and with and without replacement) to assess the effect of incarceration on prevalent asthma, diabetes, and hypertension [25,26]. Common support matching begins with defining a common support region, which excludes "treated" units whose propensity score is higher than the highest of the control units and control units whose propensity scores are lower than the lowest of the "treated" units. This process is followed by randomly selecting one control that matches on the propensity score with a "treated" participant. The advantage of this matching procedure is that it reduces the probability of a bad match, though some "treated" participants may not be matched [27]. Alternatively, matching without common support enables a match with the control with the closest propensity score, guaranteeing that a match is always found for all the "treated" units. Replacement means that once a control has been matched, it can no longer be matched. As each matching procedure has potential strengths and weaknesses, the results for all four matching estimators are presented.

#### Logistic Regressions

For medical conditions found to be significantly associated with history of incarceration, we used logistic regressions excluding and including incarceration in order to examine whether having been incarcerated mediates racial and ethnic disparities, or to ascertain the extent to which incarceration is a social pathway through which Black and Latino individuals are placed at higher odds of having that chronic condition [28]. We used a 3-step Baron and Kenny process to assess mediation: demonstrate that (1) race-ethnicity is associated with the medical condition, (2) race-ethnicity is associated with incarceration, and (3) the association of race-ethnicity is attenuated, after adjustment for incarceration [29]. As a final specification, we included the estimated propensity score of each individual - their predicted probability of having been incarcerated, in the regression model. Parameter estimates and standard errors were estimated using probability weights provided by NYC HANES to adjust for complex sampling design, non-response, and post-stratification.

## Results

Former inmates were more likely to be male, poor, less educated, and Black or Latino. They were more also more likely to report current smoking, excessive alcohol consumption and binge drinking, cocaine and intravenous drug use, and intimate partner violence compared to those never incarcerated (Table 1). We used the aforementioned variables to estimate the predicted probability of incarceration, or a propensity score.

**Table 1:** Descriptive statistics by incarceration status.

	Total % (N = 1,990)	Formerly Incarcerated % (N = 160)	Never Incarcerated % (N = 1,830)	***P***
White	38.6	26.9	39.6	.016
Black	23.0	38.3	21.6	< .001
Asian	10.8	0.3	11.8	< .001
Latino	26.0	33.1	25.4	.053
Other race	1.6	1.4	1.6	.821
				
Age 20-29	19.9	22.5	19.7	.419
Age 30-39	22.6	21.7	22.7	.779
Age 40-49	20.4	24.2	20.1	.301
Age 50-59	15.5	20.6	15.0	.124
Age 60 and older	21.5	11.1	22.5	.001
				
Male	46.0	75.9	43.3	< .001
Female	54.0	24.1	56.7	
				
Income $20,000 or more	30.9	38.4	30.3	.092
Income less than $20,000	69.1	61.6	69.7	
				
Less than HS education	26.1	35.6	25.2	.023
High school education	18.6	24.2	18.1	.122
Greater than HS education	59.1	77.6	57.4	< .001
				
Not married	55.8	66.2	54.9	.021
Married	44.2	33.8	45.1	
				
Injured by partner	2.1	7.6	1.6	.004
				
Current smoker	23.6	47.5	21.4	< .001
Former smoker	20.5	22.5	20.4	.615
Heavy drinking	38.8	46.8	38.0	.037
Binge drinking	26.0	38.6	24.8	.001
Intravenous drug user	1.4	8.8	0.8	< .001
Cocaine user	3.4	10.7	2.8	< .001

*Note*. Means and p-values adjusted for complex survey design using clinic weights for asthma and hypertension and fasting weights for diabetes. P-values are from regressing former incarceration on each of the individual background attributes, separately.

Estimates of the average treatment on treated effects of incarceration on various health outcomes are provided in Table 2. In an unmatched model (model 1), the prevalence of current asthma among formerly incarcerated individuals was higher than that among those who had never been incarcerated (12.7% vs. 6.2%, *p *< 0.001, data not shown). Using propensity score matching techniques (models 2-5), we found that having a history of incarceration is associated with asthma prevalence after controlling for the observable selection into incarceration. Depending on the modeling assumptions used in estimating the propensity scores, the difference in asthma prevalence between formerly incarcerated and never incarcerated individuals was either statistically significant at the 5% level (model 2, (13.3% vs. 5.1%), model 3 (13.4% vs. 5.2%), model 4 (13.3% vs. 4.5%) or was marginally significant at the 10% level in model 5 (13.8% vs. 4.6%), where we restricted the sample to common support and matched with replacement. We did not detect statistically significant differences in the prevalence of diabetes or hypertension between formerly incarcerated and never incarcerated individuals.

**Table 2:** Estimated Average Treatment on Treated (ATT) effects of incarceration on health.

	(1)	(2)	(3)	(4)	(5)
Sample	Unmatched	Matched	Matched	Matched	Matched
Asthma	0.065***	0.080**	0.072**	0.089**	0.092*
	(0.022)	(0.034)	(0.035)	(0.037)	(0.037)
Diabetes	-0.031	-0.013	-0.033	-0.006	-0.033
	(0.026)	(0.032)	(0.034)	(0.039)	(0.034)
Hypertension	-0.016	-0.006	-0.033	0.12	0
	(0.032)	(0.043)	(0.044)	(0.048)	(0.047)

Support	N/A	No	Yes	No	Yes
Replacement	N/A	No	No	Yes	Yes
Sample Size	1,692	1,692	1,687	1,692	1,687

*Note*. *, **, *** represent statistical significance at the 10, 5, and 1 percent levels in a two-sided t-test. Standard errors in parentheses do not take into account that the propensity score itself is estimated. Table entries are the difference in mean prevalence for each health outcome between those individuals who have been formerly incarcerated and those who have not. Parameters estimated using nearest neighbor inexact matching.

Given the association between having a history of incarceration and asthma, we examined whether having been incarcerated mediates racial and ethnic disparities in asthma prevalence. First, Blacks were more likely to have asthma as compared to White individuals (OR 1.88, 95% CI: 1.05, 3.36) even after adjustment for sociodemographics, smoking, and illicit drug use (Table 3). Second, Blacks and Latinos were more likely to have a history of incarceration compared to Whites (Blacks, OR 3.22, 95% CI 2.02, 5.19; Latinos, OR 2.36, 95% CI 1.5, 3.70, data not shown). Third, after including incarceration history in a logistic regression model, the effect of race on asthma outcomes was attenuated, indicating a partial mediation effect (OR 1.75, 95% CI: 0.98, 3.15). A formal Sobel test of mediation also confirms this finding (z-statistic 1.72, p = 0.084)[29]. Adding the propensity score in the logistic regression for Model 3 did not appreciably lessen the odds that Blacks have asthma compared to Whites. Having a history of incarceration did not affect estimates of the prevalence of current asthma among Latinos compared to their White counterparts.

**Table 3:** Estimated Adjusted Odds Ratios of Asthma.

	Model 1: Excludes Incarceration (N = 1,833)		Model 2: Includes Incarceration (N = 1,833)		Model 3: Includes Propensity Score (N = 1,833)	
			
	OR (95% CI)	***p***	OR (95% CI)	***p***	OR (95% CI)	***P***
Formerly incarcerated			2.42 (1.39, 4.20)	.002	2.42 (1.36, 4.31)	.003
						
Black^1^	1.88 (1.05, 3.36)	.033	1.75 (0.98, 3.15)	.060	1.76 (0.91, 3.39)	.091
Asian	0.73 (0.17, 3.14)	.669	0.78 (0.18, 3.38)	.733	0.77 (0.18, 3.29)	.727
Latino	1.27 (0.69, 2.31)	.438	1.25 (0.68, 2.82)	.476	1.25 (0.67, 2.33)	.487
Other race	1.57 (0.50, 4.92)	.433	1.60 (0.50, 5.08)	.426	1.60 (0.50, 5.08)	.426
						
Age 30-39^2^	0.57 (0.33, 0.98)	.041	0.56 (0.33, 0.96)	.036	0.56 (0.32, 0.97)	.039
Age 40-49	0.75 (0.42, 1.34)	.335	0.72 (0.40, 1.29)	.266	0.72 (0.40, 1.29)	.270
Age 50-59	0.68 (0.33, 1.38)	.285	0.66 (0.32, 1.36)	.262	0.66 (0.32, 1.36)	.263
Age 60 and older	0.74 (0.38, 1.45)	.377	0.77 (0.40, 1.51)	.452	0.77 (0.38, 1.56)	.469
						
Female^3^	2.35 (1.43, 3.86)	.001	2.70 (1.60, 4.56)	< .001	2.68 (1.27, 5.66)	.010
						
Income less than $20,000^4^	1.03 (0.63, 1.69)	.913	1.01 (0.62, 1.64)	.963	1.01 (0.62, 1.65)	.962
						
High school education^5^	0.61 (0.36, 1.05)	.076	0.60 (0.35, 1.03)	.065	0.60 (0.34, 1.05)	.073
Less than high school education	1.05 (0.65, 1.72)	.833	1.00 (0.61, 1.65)	.992	1.00 (0.62, 1.62)	.986
						
Married^6^	1.16 (0.70, 1.93)	.554	1.19 (0.72, 1.97)	.496	1.19 (0.70, 2.03)	.526
						
Injured by partner	0.47 (0.12, 1.93)	.293	0.40 (0.11, 1.48)	.167	0.40 (0.09, 1.73)	.218
						
Current smoker	1.73 (1.03, 2.88)	.037	1.55 (0.94, 2.58)	.088	1.56 (0.88, 2.77)	.129
Former smoker	1.42 (0.73, 2.77)	.300	1.35 (0.69, 2.65)	.380	1.35 (0.68, 2.69)	.389
Heavy drinking	1.18 (0.79, 1.75)	.413	1.13 (0.76, 1.68)	.551	1.13 (0.75, 1.71)	.560
Binge drinking	1.36 (0.89, 2.08)	.150	1.36 (0.89, 2.08)	.158	1.36 (0.89, 2.07)	.154
Needle drug user	0.29 (0.03, 2.50)	.258	0.23 (0.03, 1.94)	.176	0.24 (0.02, 2.78)	.250
Cocaine user	0.84 (0.27, 2.62)	.766	0.76 (0.25, 2.37)	.640	0.77 (0.23, 2.56)	.665
						
Propensity score					0.95 (0.18, 50.0)	.982

*Note*. OR = odds ratio; CI = confidence interval; estimates adjusted for complex survey design using clinic weight; propensity score estimated with replacement. Replacement of self-reported smoking with serum cotinine level did not change the results of this mediation analysis.

Referent group for ^1 ^race/ethnicity is white, ^2 ^age is ages 20-29, ^3 ^gender is male, ^4^income is $20,000 or more, ^5 ^education greater than high school, and ^6 ^marital status is not married.

**Figure 1:**
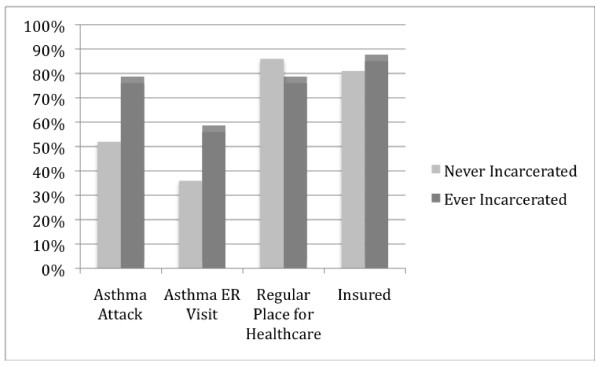
**Asthma severity symptoms and healthcare access by incarceration status among asthmatics in NYC HANES**.

Figure 1 presents the prevalence of measures of asthma severity (recent asthma attack, recent emergency department visit for asthma) and health care access (regular source of health care and health insurance status) by history of incarceration. Among asthmatics, those who reported a history of incarceration were more likely to have had an asthma attack (76% vs. 52%) or use the emergency room for asthma (56% vs. 36%) in the past 12 months and as likely to have access to a regular health care provider (76% vs. 86%) or health insurance (85% vs. 81%) compared to those never incarcerated.

## Discussion

In a population-based study of NYC adults, we found that having a history of incarceration is significantly associated with asthma prevalence. Asthma was more common among former inmates than those without this history, and this association persisted after adjustment for age, gender, self-reported race and ethnicity, smoking and illicit drug use, and measures of socioeconomic status using both standard regression and propensity score matching. We also found that asthmatics with a history of incarceration were more likely to have had an asthma attack or been seen in the emergency department in the past year, in spite of similar access to a primary care or health insurance, compared to those never incarcerated. Screening for and treating asthma during incarceration and upon release may be important in reducing asthma morbidity.

The mechanisms by which being incarcerated is associated with asthma are not known. Plausible mechanisms documented in prior studies of non-incarcerated individuals,[30] include higher rates of smoking, drug and alcohol use, domestic violence, or lower socioeconomic status among formerly incarcerated individuals[31-34]. Our data suggest that these mechanisms do not entirely explain the risk for asthma among former inmates. Other possible explanations could include dilapidated prison conditions, indoor allergens (cockroach and mouse droppings)[35], repeated bacterial or viral infections[8], and exposure to contraband and second hand smoke within correctional facilities [31]. Upon release, formerly incarcerated individuals may live in neighborhoods with substandard housing given their difficulties securing employment or public housing[36], heightening their exposure to a variety of environmental asthma triggers. Additionally, former inmates are more likely to be exposed to violence both within prison and in the community, which may increase psychological stress, thus increasing asthma symptoms and decreasing adherence with medications [37]. Finally, studies of formerly incarcerated individuals with tuberculosis and HIV have demonstrated that adherence to medications worsens after release from prison, which may also be a contributor to worse asthma outcomes observed in this study [38]. Additional studies are warranted to investigate the specific mechanisms by which formerly incarcerated individuals may have increased odds for worse asthma outcomes.

Additionally, our results suggest that having a history of incarceration plays a role in augmenting Black-white disparities in asthma prevalence. Two plausible scenarios could explain this finding. First, because Blacks are significantly more likely to be incarcerated, even if the effect of incarceration on health is similar across races, its impact is greater on the aggregate health of Blacks because they are more likely to be incarcerated. Alternatively, exposure to the criminal justice system may be worse for minorities (worse prison housing, longer sentences)[3], thus differentially augmenting the prevalence of asthma among Blacks compared to their White counterparts.

Although exploring the mechanisms whereby incarceration may contribute to racial disparities in asthma is beyond the scope of the present study, our data raise the importance of additional studies exploring how incarceration may contribute to racial health disparities. To this end, local population-based studies might consider enrolling institutionalized adults or at least consider adding questions about past encounters with the criminal justice system, such that this relationship can be further studied longitudinally. Additionally, studies centered on eliminating racial health disparities which censor participants upon incarceration might consider following individuals into correctional facilities where permitted, in order to understand how being incarcerated differentially affects disease management and access to health care among minority populations [39,40].

Finally, detention in jail or prison, where health care is constitutionally guaranteed, may present a prime opportunity to engage minority populations in disease prevention and treatment and reduce racial disparities. The best known models for bridging health care from correctional facilities to the community involve health care providers and case managers that work both in the correctional facilities and in the community to ensure continuity of care [41,42]. Although effective in smaller geographic settings, these programs may be difficult to replicate in correctional systems that release prisoners to distant communities. Communities with larger state correctional systems are turning to other programs, which engage individuals immediately prior to or after release from prison using community health workers and utilize the existing structure of the safety net public health system to transition individuals back to community care [43]. Reaching the 2010 Healthy People goal of eliminating health disparities should include an improved understanding of both the role incarceration and the criminal justice system in augmenting racial and ethnic health disparities and its potential as an intervention point for reducing disparities.

There are several limitations in our study. NYC HANES is a cross-sectional study; as such, we are unable to attribute causality. The study also exclusively recruited housed, non-institutionalized NYC residents, selecting for individuals who are less likely to have been incarcerated. As such, we may not have had the power find an association between incarceration and three different chronic medical conditions, thus raising the possibility of type II error as the sample size was limited. Moreover, incarceration was only measured as a single binary question item capturing events that would have included both a brief jail stay after arrest as well as longer periods of imprisonment, thus likely diluting any effect we did detect. Finally, the choice of variables used in the construction of the propensity scores was limited by those measured in NYC HANES. For instance, we did not have measures of neighborhood effects, which have been shown to be related to the prevalence of asthma, diabetes, hypertension [44]. Nonetheless, our propensity score did include variables most likely to be important confounders in the relationship between incarceration and asthma, diabetes, and hypertension--namely measures of race/ethnicity, socioeconomic status, smoking, alcohol use, and illicit drug use.

## Conclusion

For this study, we chose to examine the association between having a history of incarceration and three prevalent chronic medical conditions for which there are documented racial and ethnic disparities in NYC--asthma, diabetes, and hypertension. Using data from NYC HANES, one of the few population-based studies on chronic disease which asks participants about incarceration history, we found that having been incarcerated may augment Black-white disparities in asthma among NYC residents. This study demonstrates how including measures of incarceration in epidemiologic studies can improve our understanding of the health of racial and ethnic minorities with asthma. Reaching our national goal of eliminating racial health disparities warrants a better understanding of the role that incarceration and the criminal justice system may play in augmenting these disparities and how prisons and jails might be a suitable place for intervention.

## Competing interests

The authors declare that they have no competing interests.

## Authors' contributions

EAW conceived of the study, participated in its design and coordination, performed statistical analysis, and drafted the manuscript. JG participated in the design of the study, performed the statistical analysis, and helped to draft the manuscript. All authors read and approved the final manuscript.

## Pre-publication history

The pre-publication history for this paper can be accessed here:

http://www.biomedcentral.com/1471-2458/10/290/prepub
